# Thermodynamic Properties and State Diagram of Gum Ghatti-Based Edible Films: Effects of Glycerol and Nisin

**DOI:** 10.3390/polym12020449

**Published:** 2020-02-14

**Authors:** Pingping Zhang, Ya Zhao, Xin Zhang, Lanlan Zhu, Zhongxiang Fang, Qilong Shi

**Affiliations:** 1 School of Agricultural Engineering and Food Science, Shandong University of Technology, Xincun West Road, Zibo, Shandong 255000, China; 2 School of Agriculture and Food, The University of Melbourne, Parkville 3010, Australia

**Keywords:** gum ghatti, glycerol, nisin, thermodynamic properties, state diagram

## Abstract

In this present study, the thermodynamic and thermal properties of glycerol and nisin-incorporated gum ghatti (GG, *Anogeissus latifolia*)-based films were determined. The films exhibited type III isotherm behaviors. Moisture content (MC) of films was increased with increasing water activity (*a*_w_) and decreased with higher temperature. The incorporation of glycerol and nisin increased the sorption ability of GG films. The net isosteric heat of adsorption (*q*_st_) and differential entropy (*S*_d_) were decreased with increasing MC, showing an exponential negative correlation between them. Spreading pressure (*φ*) was increased with increasing *a*_w_, but decreased with higher temperature. This incorporation of glycerol and nisin increased the *q*_st_, *S*_d_ and *φ* of the GG films. The sorption behaviors were enthalpy-driven and non-spontaneous processes. The glass transition temperature (*T*_g_), critical MC and *a*_w_ of the films were decreased, and increased respectively with the incorporation of glycerol and nisin. This work provides a theoretical basis for the application of edible films in fresh food preservation.

## 1. Introduction

Numerous researches have been performed recently in the development of edible and biodegradable packaging materials from natural biopolymers because of consumers’ increased health and environmental concerns induced by synthetic plastic [1,2]. Gum ghatti (GG) is the sap collected from the tree *Anogeissus latifolia/indifolia*, which is mainly grown in India [3]. In a previous study, Zhang, Zhao and Shi [4] suggested that GG has a great potential in edible film fabrication with anticipated features, such as an excellent barrier, along with mechanical, optical and structural properties. 

As a packaging material, edible films’ ability to restrict the moisture transfer between the food and its surrounding environment is an important attribute [5]. The adsorbed water will interfere with the films’ physical and mechanical characteristics and give rise to considerable performance deterioration when the moisture content (MC) of films reaches a critical value [6,7]. Therefore, it is extremely important to reveal the water change rules under various ambient conditions, such as temperature and relative humidity (RH). Water activity (*a*_w_) implies the ‘quality’ of water content, and exhibits the bound degree of water, which is important to evaluate the availability of water involved in physical, chemical and microbial reactions [8]. The moisture sorption isotherm (MSI) reveals the relevance of equilibrium moisture content (EMC) and *a*_w_ or equilibrium-relative humidity (ERH) under a specific temperature and pressure [8]. Moisture thermodynamics are derived from isotherm data and regarded as a dependable guideline to estimate physical properties and the storage stability of foods [9,10]. 

Thermodynamic parameters include the net isosteric heat of sorption (*q*_st_), differential entropy (*S*_d_), spreading pressure (*φ*), and the enthalpy–entropy compensation theory [6,7]. The *q*_st_ value implies the binding strength between water and food solid (by intermolecular attraction forces between sorbate and adsorptive sites); *S*_d_ is proportional to the number of available adsorption sites at a determined energy level; *φ* or surface potential signifies the excess free energy of the surface, and means the increase of surface tension of bare adsorption sites as a result of adsorbed molecules; enthalpy–entropy compensation theory (also termed as the isokinetic theory) is useful to acquire information concerning the mechanisms that dominate the moisture adsorption process [9,10].

Glass transition theory is commonly used to describe the relationship of biopolymers’ structure and its properties, as well as their relevance to food processing and preservation [5]. The glass transition theory is of vital importance to the edible film field, since the films’ mechanical and barrier characteristics are significantly affected by the glass transition temperature (*T*_g_) of the film matrix [11]. State diagram, the most valuable application of *T*_g_, is graphical explanations of the physical states of food ingredients in regard to temperature and the moisture/solid content at a determined pressure, and it can be adopted to reveal the complex physical and chemical variations that emerged in foods, and select suitable processing/storage conditions of food systems [12,13]. 

In biopolymer film preparation, plasticizers are usually added to interact with the polymer chains, which are competent for orienting them between the chains of polymers, and interfering with the interactions between polymers accompanying the improvement of molecular mobility, flexibility and processability [14]. To satisfy consumers’ demand for the high efficiency of food preservation, active antimicrobial packaging films have been developed [2,15]. Antimicrobial films are commonly achieved by adding antimicrobial agents (such as nisin) into films, forming matrices [16,17]. However, the performance for hydrophilic films are markedly altered owing to the plasticization effects, either when the films are placed under different values of RH during transportation and storage, or the films was incorporated with plasticizers and antimicrobial agents [18]. However, little attention has been paid to the thermodynamic properties and state diagram of edible films, especially GG-based films. Therefore, the purposes of this work were to investigate the effect of glycerol and nisin on the adsorption characteristics and thermodynamic properties, together with the state diagram of films, and evaluate the performance of GG-based films, utilizing critical parameters, viz. critical *a*_w_ and critical MC, based on the state diagram.

## 2. Material and Methods

### 2.1. Materials

Gum ghatti, glycerol and nisin were acquired from Shanghai Quanwang Biotechnology Co. Ltd. (Shanghai, China). Inorganic salts used for relative humidity (RH) control include LiCl, CH_3_COOK, MgCl_2_, K_2_CO_3_, Mg(NO_3_)_2_, NaNO_2_, NaCl, KCl and BaCl_2_, which were of analytical grade and obtained from Tianjin Reagent Chemicals Co. Ltd. (Tianjin, China). 

### 2.2. Preparation of Films 

Pure gum ghatti (GG) film (GG-P), GG film with the incorporation of glycerol (GG-G), and GG film with an incorporation of glycerol and nisin (GG-G-N) were prepared according to Zhang et al. [4] and Zhao, Zhang and Shi [19]. Briefly, aqueous solutions of 0.75% GG were prepared under 15-min constant stirring at ambient temperature. GG molecules were dispersed and fully hydrated using a disperser (Ultra-Turrax, IKA T18, IKA-Werke Gmbh & Co. KG, Staufen, Germany). The solution was kept at 40 °C for 60 min in a thermostatic water bath. After that, film-forming solution was divided into three equal parts: (1) a control GG-P solution without the incorporation of plasticizer and antimicrobial agent; (2) a GG-G solution with addition of 30% (*w*/*w*, based on GG weight) glycerol into the GG solution; (3) a GG-G-N solution with addition of 10% (*w*/*w*, based on GG weight) nisin into the GG-G solution. To remove air bubbles, the three film-forming solutions were then put into an ultrasonic apparatus (SK3310LHC, Kudos Ultrasonic Instrument Co., Ltd., Shanghai, China) for 60 min. Finally, the solutions were respectively casted on Petri dishes and heat pump dried (1HP-5, Qingdao Oumeiya Technology Co. Ltd., Qingdao, China) at 25 °C for 24 h. The resulting films were peeled off from the casting surface.

### 2.3. Moisture Sorption Isotherms

Triplicate film samples were placed into airtight desiccators and equilibrated with various saturated salt solutions to obtain *a*_w_ ranging from 0.113 to 0.931 [20,21], and the values are shown in Table 1. The desiccators were put into incubators with set temperatures (viz., 5, 15 and 25 °C). Weighing the samples regularly until constant weight was attained. Moisture content of the samples was obtained by drying in an electric oven (DHG-9140A, Yiheng Scientific Instrument Co., Ltd., Shanghai, China) at 90 °C for 24 h [4].

The moisture sorption isotherm data of GG-based films were fitted to Guggenheim-Anderson-De Boer (GAB) (Equation (1)) and the Halsey model (Equation (2)) [22].

(1) $$X=\frac{X_{m}{}_{g}C_{g}Ka_{w}}{\left(1-Ka_{w})(1-Ka_{w}+C_{g}Ka_{w}\right)}$$

(2) $$X={\left(\frac{-a}{\ln a_{w}}\right)}^{\frac{1}{r}}$$

where *X* is moisture content (dry basis, d.b.); *X_mg_* is the GAB monolayer moisture content; *C_g_*, *K*, *a* and *r* are constants.

The model fit precision was judged by the coefficient of determination (*R*^2^) and the standard error of estimate (*SE*) between the experimental and predicted data using Equation (3) [13].

(3) $$SE=\sqrt{\frac{\sum_{i=1}^{N}{\left(X_{e,i}-X_{p,i}\right)}^{2}}{N-n}}$$

where *N* and *n* are the number of experimental observation and the number of constants in each model, respectively; *X*_e,i_ and *X*_p,i_ are the experimental and predicted moisture contents, respectively. 

### 2.4. Thermodynamic Properties

#### 2.4.1. Net Isosteric Heat of Sorption (q_st_) and Differential Entropy (S_d_)

The *q*_st_ can be deduced from sorption isotherm data by Clausius–Clapeyron equation [23]:

(4) $$q_{st}=-R{\left(\frac{d\ln a_{w}}{d(1/T)}\right)}_{X}$$

where *R* is the universal gas constant, 8.314 J/(mol·K); *T* is the sorption temperature (*K*); *a*_w_ is water activity; *X* is moisture content (d.b.). 

By plotting ln *a*_w_ versus 1/*T* at determined moisture content, then *q*_st_ can be calculated from the slope.

The relationship between *q*_st_ and *S*_d_ is given by Equation (5) [24].

(5) $${\ln a_{w}|}_{X}=-\frac{q_{st}}{RT}+\frac{S_{d}}{R}$$

By plotting ln *a*_w_ versus 1/*T* at constant moisture content, then *S*_d_ can be calculated from the intercept.

#### 2.4.2. Enthalpy-Entropy Compensation Theory

The compensation theory proposes a linear relationship between *Q*_st_ and S_d_ [25]:

(6) $$Q_{st}=T_{\beta }S_{d}+\Delta G_{\beta }$$

where *Q*_st_ is the isosteric heat of sorption, the value of which is *q*_st_ plus the heat of vaporization of water at the system temperature; *T*_β_ is the isokinetic temperature (*K*), representing the temperature at which all the reactions proceed at the same rate; Δ*G*_β_ is the Gibbs’ free energy at *T*_β_ (J/mol). 

By plotting *Q*_st_ against *S*_d_, *T*_β_ and Δ*G*_β_ can be obtained by linear regression. 

Comparison between *T*_β_ and harmonic mean temperature (*T*_hm_) can be used to verify the validity of the compensation theory; and the compensation theory exists only when *T*_β_ ≠ *T*_hm_ [26]. The sorption process is enthalpy-driven if *T*_β_ > *T*_hm_, and is entropy-driven if *T*_β_ < *T*_hm_ [27]. *T*_hm_ is defined as:

(7) $$T_{\mathrm{hm}}=\frac{n}{\sum_{i=1}^{n}(1/T_{i})}$$

where *n* is the number of sorption isotherms employed.

#### 2.4.3. Spreading Pressure (φ)

The *φ* was determined using the equation described by Moreira et al. [23]:

(8) $$\varphi =\frac{K_{B}T}{A_{m}}a^{\frac{1}{r}}{\left(\frac{1}{(\frac{1}{r}-1){\left(-\ln a_{w}\right)}^{\frac{1}{r}-1}}\right)}_{0.05}^{a_{w}}$$

where *K*_B_ is Boltzmann’s constant (1.38 × 10^−23^ J/K); *A*_m_ is the surface area of a water molecule (1.06 × 10^−19^ m^2^); *a* and *r* are the constants of Halsey equation (Equation (2)).

### 2.5. Glass Transition Temperature (T_g_)

The *T*_g_ of films equilibrated at different RHs was analyzed using a differential scanning calorimetry (DSC) (Q100, TA Instruments, New Castle, DE, USA). A single scanning program was adopted for the analysis of *T*_g_ [13]. Briefly, samples (5~10 mg) were enclosed in hermetically sealed aluminum pans and placed on the DSC. The sample was scanned between −100 and 200 °C at 10 °C/min. The midpoint of the glass transition was chosen as the characteristic’s temperature of transition. 

The relationship between the *T*_g_ and water of GG-based films can be described by the Gordon–Taylor equation [13].

(9) $$T_{g}=\frac{(1-X_{w})T_{gs}+kX_{w}T_{gw}}{(1-X_{w})+kX_{w}}$$

where *T*_g_, *T*_gs_ and *T*_gw_ are the glass transition temperature of film samples, film solids and water, respectively; *X*_w_ is the mass fraction of water; *k* is the model parameter.

### 2.6. Statistical Analysis

Model parameters were achieved by non-linear regression analyses (Matlab 7.0 software, MathWorks Corp., Natick, MA, USA). The analysis of variance (ANOVA) was carried out by SPSS software (Version 17.0, SPSS Inc., Chicago, IN, USA) and Duncan′s multiple range test was selected to evaluate the significant differences (*p* < 0.05).

## 3. Results and Discussion

### 3.1. Moisture Sorption Isotherm

**Figure 1:**
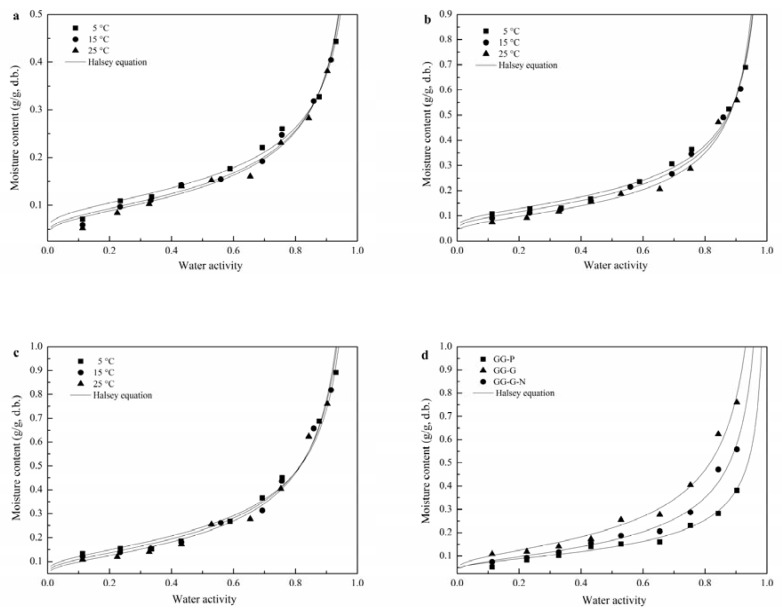
Moisture sorption isotherms of gum ghatti (GG)-based films at different temperatures; (**a**) GG-P, (**b**) GG-G, (**c**) GG-G-N, and (**d**) temperature of 25 °C. GG-P, pure gum ghatti film; GG-G, gum ghatti film with incorporation of glycerol; GG-G-N, gum ghatti film with incorporation of glycerol and nisin.

Figure 1 shows the isotherm plots at 5, 15 and 25 °C, from which the effect of plasticizer (glycerol) and antimicrobial agent (nisin) on the sorption characteristics of GG-based films was observed. GG-based films followed typical type III sorption behaviors. The EMC was increased with increasing *a*_w_ at a given temperature, which can be attributed to the films’ tendency of lowering the vapor partial pressure as the RH of ambient air was decreased [28]. The EMC of GG-based films was decreased with higher temperature at determined *a*_w_, as shown in Figure 1a–c. This tendency could be interpreted by the activation state of molecules. The molecule–molecule attractive forces are decreased with increasing temperature, owing to an increase in the kinetic energy, which results in an increased mutual distance of water molecules [9]. Consequently, water molecules became less stable, and easily run away from the binding sites [7]. It was noted that at the employed temperature range (viz. 5–25 °C), a tendency to crossover at *a*_w_ > 0.85 can be observed from the MSI of GG-based films. The crossover behavior during the moisture sorption of polymers is mainly attributed to the over-exposure of the active sites and/or hydrophilic groups under higher *a*_w_ conditions with the increase of temperature [29]. Similar observations have been reported for MSI of pure alginate films [7]. 

In Figure 1d, the EMC of all three films was slowly increased until *a*_w_ approaches 0.60, afterwards a marked increase in EMC was observed, especially for films with glycerol and nisin (GG-G-N). At a constant *a*_w_ and temperature, pure GG film had the lowest EMC, whereas an addition of glycerol and nisin increased the film moisture sorption capacity. This trend was more obvious especially at *a*_w_ > 0.60. This might be due to the dual plasticization effects of glycerol and the water molecule. This phenomenon also implied that glycerol was more hydrophilic than nisin. The addition of glycerol to GG films enhanced the hygroscopic nature of the film, which can be attributed to the low molecular weight and polar solute characteristics of glycerol [5]. However, the reason for the increased hygroscopic nature of GG-G-N might be due to the exposure of hydrophilic groups during film formation and moisture sorption procedures.

Fitting the sorption isotherm models to the experimental data of GG-based films is shown in Table 2. As can be observed, the GAB and Halsey models correlated well to the water sorption isotherm data over the whole range of temperatures, and *a*_w_ investigated due to higher *R*^2^ and lower *SE* values. However, it should be noted that merely higher *R*^2^ and lower *SE* values are not enough to guarantee the statistically validity of model fitting. Residual plots were then analyzed at different temperatures. Among the selected models, the Halsey model provided a scattered or random residual pattern (Table 2). Therefore, the Halsey model is suitable for expounding the sorption behavior for GG-based films at temperature and the *a*_w_ range of 5–25 °C and 0.113–0.931, respectively.

### 3.2. Thermodynamic Properties

#### 3.2.1. Net Isosteric Heat of Sorption and Differential Entropy

**Figure 2:**
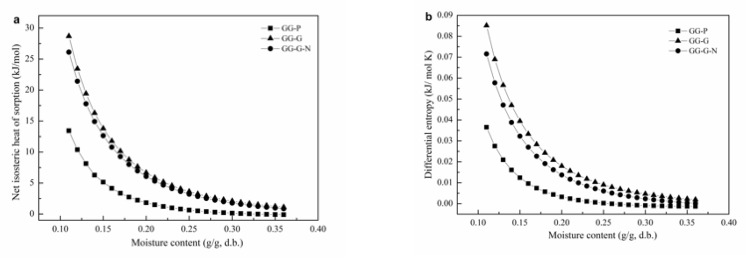
Net isosteric heat of sorption (**a**) and differential entropy (**b**) for GG-based films as a function of moisture content.

The variations of *q*_st_ versus the moisture content of GG-based films are shown in Figure 2a. The *q*_st_ decreased acutely at moisture content < 0.15 g/g, and relative slowly at moisture content ranging from 0.15 to 0.25 g/g. The decrease of *q*_st_ with increasing moisture content is a symbol of weak interactions between water and the surface of films. At a low moisture content level (e.g. in the monolayer region), water can be absorbed at the strongest binding sites on the film surface. With the increase of moisture content, the binding sites become occupied, and sorption occurs on the less available sites with lower binding energies [7]. The *q*_st_ approaches to 0 as the moisture content > 0.25 g/g, which indicates that sorption heat is nearly the same to the vaporization heat of free water [6]. At a low moisture content level (e.g., < 0.15 g/g), the highest *q*_st_ values were observed for film GG-G, followed by GG-G-N and GG-P. The data imply that water molecules were strongly interacted with the sorption sites of GG-G and GG-G-N, compared with GG-P. However, the differences in *q*_st_ values for GG-G and GG-G-N became negligible with increasing moisture content (Figure 2a). Furthermore, the binding forces are similar for all GG-based films when the moisture content was higher than 0.35 g/g. The value of *q*_st_ at a specific moisture content provides an indication of the state of the adsorbed water, and therefore a measure of the physical, chemical and microbiologic stability of the food material at given storage conditions. Furthermore, information about energy consumption and drying equipment design can also be obtained from the changes of the *q*_st_ with moisture content [9].

Figure 2b shows the variation of *S*_d_ as a function of moisture content for GG-based films. Similar to the pattern of the *q*_st_, a strong dependence upon moisture content with an exponential trend are displayed for the *S*_d_ of GG-based films. The *S*_d_ decreased sharply to a moisture content of 0.15 g/g, and achieved a plateau when the moisture content continues to increase. The *S*_d_ value of GG-G and GG-G-N were higher than that of GG-P, irrespective of the moisture content. However, as moisture content became higher than 0.35 g/g, the differences significantly shortened. It is well known that sorption entropy is in proportion to the number of sorption sites at a specific energy level [30]. This work suggested that incorporation of glycerol and nisin can increase the number of available sorption sites of GG-based films.

#### 3.2.2. Enthalpy-entropy Compensation Theory 

Enthalpy–entropy compensation theory (isokinetic theory) is commonly employed to evaluate polymers’ physical and chemical phenomenon such as moisture sorption [24,27]. This theory is of vital importance to assess whether there is greater molecular interaction or to the link of the molecules in the system; enthalpy-driven means the matrix forms larger organization or order over disorganization, whereas entropy-driven implies there is greater freedom of the molecules in the food [31]. This theory illustrates that compensation phenomenon emerges due to variations in the sorbate–sorbent interactions. The enthalpy–entropy relationships of GG-based films are shown in Table 3, in which a linear relation between these variables was observed with a high regression coefficient (*R*^2^ = 0.999), implying the applicability of the enthalpy–entropy compensation theory for the sorption behaviors of GG-based films in the investigated moisture contents. The *T*_β_ values calculated from Eq. (7) were 359.3, 332.4 and 355.2 K for the sorption of GG-P, GG-G and GG-G-N, respectively. However, a linear relationship between *q*_st_ and *S*_d_ does not necessarily mean that the compensation theory is applicable. To determine the validity of the compensation theory, the *T*_hm_ value was calculated and compared with the *T*_β._ The compensation theory exists, provided that *T*_hm_ is significantly different from *T*_β_ [26]. The value of *T*_hm_ (297.9 K), which is significantly (*p* < 0.05) different from the values of *T*_β_, verifies the applicability of the compensation theory for the moisture sorption of GG-based films. In addition, the moisture sorption process for all tested films within the examined moisture ranges was considered as enthalpy–driven, because the *T*_β_ values were much higher than the *T*_hm_ value. It was reported that enthalpy-controlled behaviors depend upon food ingredients; nevertheless, entropy-controlled behaviors rely mainly on the microstructure of the matrix [22]. These observations imply that the microstructure of the GG-based films was stable in the process of moisture sorption within the temperature range from 5 to 25 °C. Furthermore, the addition of glycerol and nisin merely altered the chemical composition of GG film, and did not alter its microstructure.

Furthermore, since Δ*G* can be used as a symbol of the sorbent–water affinity, it also provides a rule concerning whether the water sorption behavior is a spontaneous or non-spontaneous feature [7]. The Δ*G* values of GG-P, GG-G and GG-G-N were 535.0, 598.6 and 1031.6 kJ/mol, respectively. These results indicated that the moisture sorption processes of GG-based films were non-spontaneous, suggesting that the GG-based films do not absorb water from the environment spontaneously, which is desirable because the product is stable [25]. Cefalas et al. [32] and Gavriil et al. [33] discovered that an entropic potential alternation competes with a thermodynamic potential from the electric dipole attachment of molecular adsorbates in polymeric ligands and the power of an associated entropic nanothermodynamic potential is proportional to the number of nanocavities induced by photon treatment. Therefore, to properly understand the water sorption mechanism, both enthalpy–entropy compensation and competition theory should be considered in future research. 

#### 3.2.3. Spreading Pressure 

The force parallel to the surface that should be imposed perpendicular to each unit length of edge to prevent the surface from spreading is termed as *φ* [22]. It is reported that high *φ* values indicate a high-water molecule-active site affinity, and the more hygroscopic the product is, the higher value the *φ* will be [25,34].

**Figure 3:**
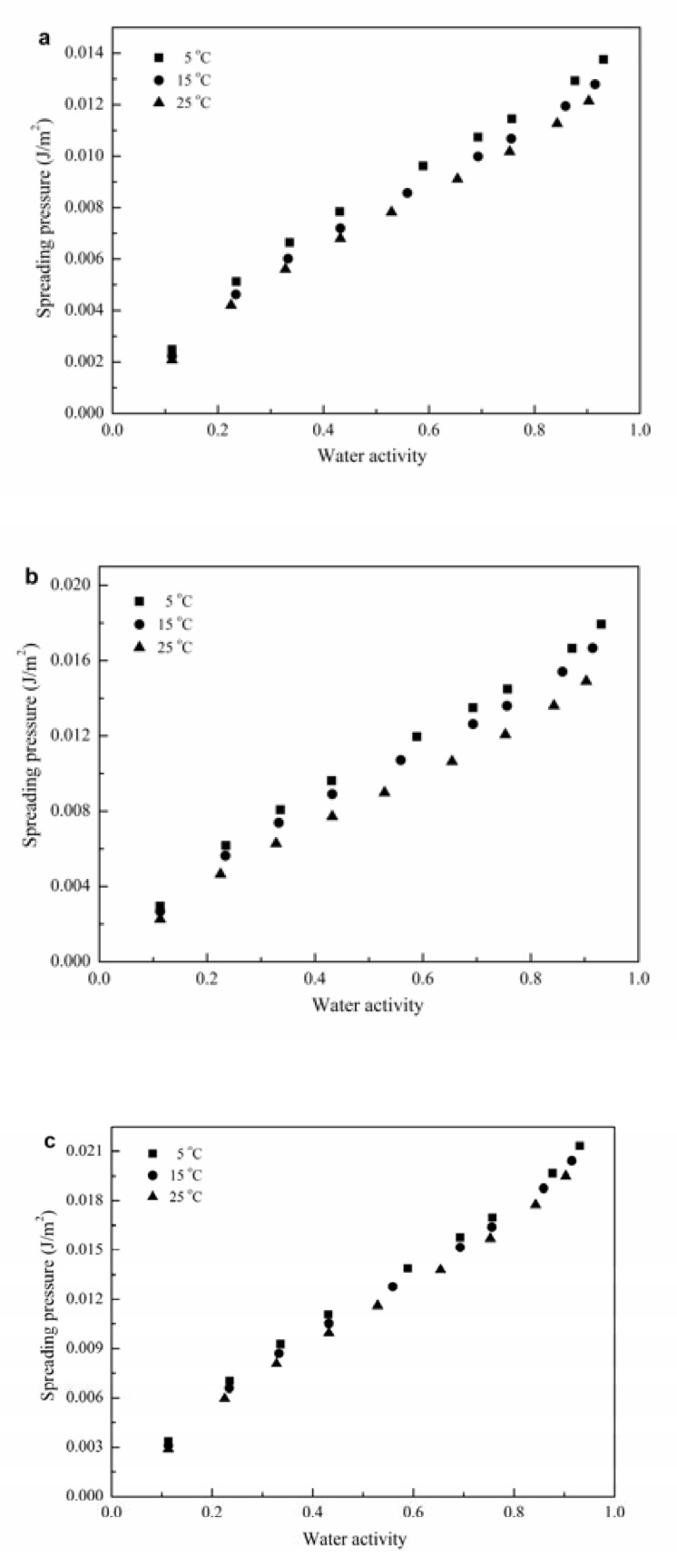
Spreading pressure as a function of water activity of for GG-based films at different values of water activity; (**a**) GG-P, (**b**) GG-G, and **(c**) GG-G-N.

The *φ* of isotherms of GG-based films at different temperatures and *a*_w_ are shown in Figure 3, where the *φ* was increased with increasing *a*_w_ and decreased with increasing temperature at a specific *a*_w_. Similar trends have been reported in pullulan–sodium alginate-based films [7]. The *φ* values of GG-G and GG-G-N were higher than those of pure GG films. This might be owing to the modifications in the conformation and/or topology of the GG molecules and/or the ratio of hydrophilic/hydrophobic sites adsorbed at the interface of GG-based films caused by incorporation of glycerol and nisin. 

### 3.3. Glass Transition Temperature (T_g_)

**Figure 4:**
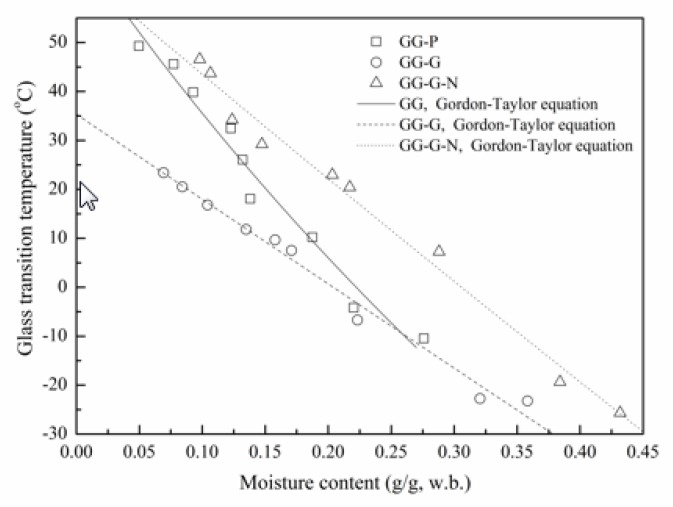
Effect of moisture content on the glass transition temperature of GG-based films.

Figure 4 shows the fluctuation of *T*_g_ as a function of moisture content for GG-based films. The *T*_g_ was decreased with increasing moisture content for all GG-based films. *T*_g_ of GG-P was decreased linearly from 49.3 to −10.5 °C as the moisture content increased from 0.050 to 0.276 g/g. However, for GG-G-N film, *T*_g_ was decreased linearly from 46.6 to −25.7 °C as the moisture content increased from 0.098 to 0.432 g/g. The anticipated decrease of *T*_g_ with the increasing moisture content can be attributed to the plasticization effect of water, which signifies a decrease in the mean molecular weight in the food system and the corresponding increase in the molecular mobility of the amorphous constituents in the matrix [5]. The *T*_g_ of food polymers depend mainly on the water content, food ingredients and mean molecular weight of the solutes existed in the food system [12]. Therefore, the *T*_g_ of polymers is decreased by the incorporation of low molecular weight components and increased by the incorporation of high molecular weight additives compatible with the food solids. As moisture content is close to 0.220 g/g, the *T*_g_ values for GG-P, GG-G and GG-G-N were −4.2, −6.7 and 20.5 °C, respectively. This was a reasonable due to the plasticizer characteristics of glycerol, which decreased the mean molecular weight in the system and influenced the molecular mobility and mechanical characteristics of the films [5]. However, for the GG-G-N films, the anti-plasticization effect of nisin was observed, which increased the mean molecular weight in the matrix and decreased the molecular mobility, and affected the mechanical properties of the films.

The *T*_g_ data of plasticized films was employed to fit the Gordon–Taylor equation and the *T*_gs_ values for GG-P, GG-G and GG-G-N were found to be 71.5, 31.0 and 63.6 °C, respectively. The prominent declining effect of glycerol on *T*_g_ was observed. However, with the incorporation of nisin, the *T*_g_ of GG-G was increased from 31.0 to 63.6 °C. The parameter *k* means the strength of the biopolymer–water interaction. For a binary mixture, a larger *k* indicates that the solids are more easily plasticized by water than food mixtures with a smaller *k* and equivalent water content [12]. The *k* value of GG-P was decreased with the addition of glycerol and nisin in the film-forming solution. The *k* values for GG-P, GG-G and GG-G-N were 1.99, 0.93 and 1.12, respectively. The *k* value was the smallest in the merely glycerol-containing film (GG-G), but was increased when nisin was incorporated in the GG-G film-forming solutions. The results showed that nisin interacted with the GG-G matrix and modified the water plasticization behavior. A similar trend has been reported by Fabra et al. [5] for sodium caseinate films as affected by lipid (oleic acid and beeswax).

### 3.4. State Diagram of GG-Based Films 

**Figure 5:**
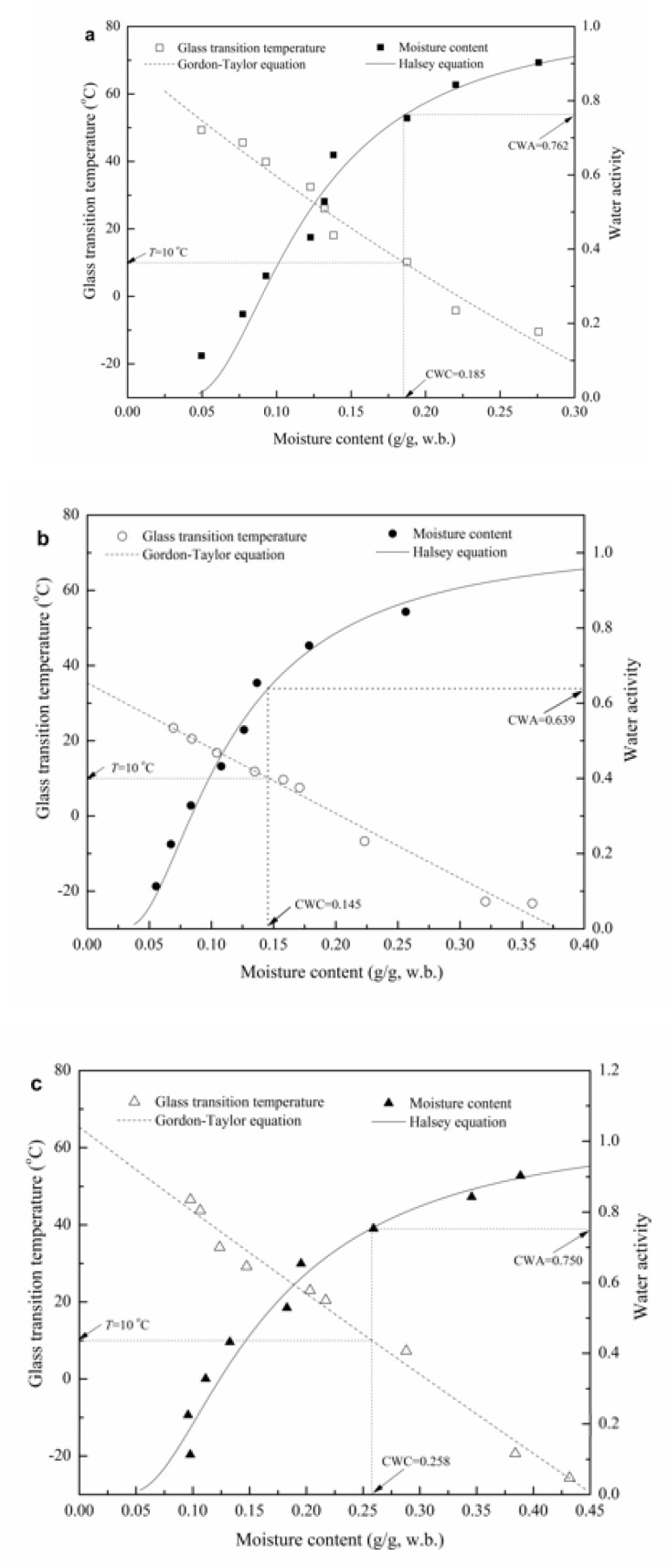
Variation of glass transition temperature, water activity with moisture content of GG-based films; (**a**) GG-P, (**b**) GG-G, and (**c**) GG-G-N.

The state diagram describes the physical state as well as state transitions of foods, which can be chosen as valuable tools in selecting the suitable temperature and moisture content condition for their manipulation and storage [35]. Recently, water activity and glass transition theories are combined to provide a unified stability evaluation criterion for foods [35]. Therefore, the sorption isotherms and *T*_g_ of GG-based films were merged to evaluate the critical parameters (viz. CWC and CWA) of films (Figure 5a). At the temperature of 10 °C (assuming this is the temperature used for the storage of GG-based film packaged foods), the CWC for the glass transition of GG-P, calculated from the Gordon–Taylor equation, was 0.185 g/g. The corresponding CWA was then acquired from the Halsey equation, and the value was calculated to be 0.762, which suggested that the maximum ERH that could ensure the glassy state of the films throughout the storage is 76.2%. Similarly, the CWC and CWA for GG-G and GG-G-N at 10 °C were calculated to be 0.145, 0.258 g/g and 0.639, 0.790, respectively (Figure 5b,c). This outcome suggested that at the same storage and/or transport temperature, the CWC and CWA of pure GG film was decreased with the incorporation of glycerol, and increased with the incorporation of nisin. If the GG-based films are applied for the packaging of fresh foods (such as fruits, vegetables and aquatic products), the CWC and ERH of pure GG film that guarantee the maximum mechanical and physicochemical properties might decrease for GG-G films and increase for GG-G-N films. That is, the storage stability/shelf life of GG-G-N packaged fresh foods is the highest; whereas GG-G packaged fresh foods possess the lowest shelf life compared with the GG-P and GG-G-N packaged pattern. Using a more specific example to explain: If the perishable foods (such as fruits and vegetables) are packaged by GG-G films and stored at 10 °C, and the ERH is controlled at 90%, which is much higher than the CWA of GG-G films. Under this circumstance, the film is in the rubbery state which promotes the molecular mobility, accelerates the permeability and reduces mechanical properties, and therefore leads to decreased perseveration [6,36]. The increased CWA and CWC of GG-G-N imply a potentially improved preservation effect when the films are employed in edible film packaging. The state diagram constructed in this study can be employed to assess the relationship between the *T*_g_ and some physicochemical and mechanical properties (such as oxygen and water vapor permeability, tensile strength, elongation at break, etc.) of films during dehydration (films making) and utilization as packaging materials (films application) for perishable foods (such as fruit and vegetables, aquatic products, etc.).

## 4. Conclusions

The MSI of GG-based films followed typical type III isotherms. Compared with GG-P, the incorporation of glycerol and nisin increased the sorption ability of films. The *q*_st_ and *S*_d_ were deceased markedly up to a moisture content of 0.15 g/g, and arrived at a steady state as the moisture content further increased. The *φ* was increased with increasing *a*_w_ and decreased with higher temperature. Compared with GG-P, an incorporation of glycerol and nisin increased the *q*_st_, *S*_d_ and *φ* of films, especially at moisture content below 0.15 g/g. The sorption mechanism in GG-based films was enthalpy-driven and non-spontaneous. The plasticization of GG-based films by glycerol and adsorbed water molecules on polymeric chains were observed. 

The state diagram of films revealed that at a determined temperature, the CWC and CWA of GG-P were decreased with the incorporation of glycerol and increased with the incorporation of nisin. To have a more in-depth understanding on water thermodynamic and thermal properties, and to investigate their effects on the physicochemical properties of packaged foods, further research should be carried out on the effects of moisture content and/or the internal environmental ERH of films on their physicochemical, mechanical and microstructure properties at different temperatures, such as *T* >> *T*_g_, *T* ≈ *T*_g_, *T* << *T*_g_.

## Figures and Tables

**Table 1:** Water activity of selected saturated salt solution at 5, 15 and 25 °C.

Saturated Salt Solutions	Temperature (°C)		
	5	15	25
LiCl	0.113	0.113	0.113
CH_3_COOK	0.235	0.234	0.225
MgCl_2_	0.336	0.333	0.328
K_2_CO_3_	0.431	0.432	0.432
Mg(NO_3_)_2_	0.589	0.559	0.529
NaNO_2_	0.693	0.693	0.654
NaCl	0.757	0.756	0.753
KCl	0.877	0.859	0.843
BaCl_2_	0.931	0.915	0.903

**Table 2:** Estimated parameters of the selected models fitted to sorption data of GG-based films at different temperatures.

Model	Parameter	Temperature (°C)								
			5			15			25	
		GG-P	GG-G	GG-G-N	GG-P	GG-G	GG-G-N	GG-P	GG-G	GG-G-N
	*X*_mg_ (g/g)	0.080	0.118	0.133	0.086	0.111	0.127	0.080	0.096	0.127
	*C*_g_	20.00	24.23	23.41	21.67	16.51	31.91	19.90	15.62	13.72
GAB	*K*	0.87	0.92	0.93	0.86	0.90	0.93	0.87	0.92	0.92
	*R* ^2^	0.985	0.995	0.992	0.993	0.996	0.991	0.985	0.984	0.988
	*SE*	0.0151	0.0170	0.0258	0.0106	0.0131	0.0264	0.0246	0.0299	0.0299
	Residual pattern		Scattered		Scattered	Patterned	Scattered		Scattered	
	*a*	0.013	0.0380	0.062	0.016	0.040	0.068	0.017	0.044	0.072
	*r*	2.148	1.830	1.709	1.942	1.709	1.587	1.836	1.537	1.493
Halsey	*R* ^2^	0.989	0.991	0.988	0.989	0.989	0.987	0.985	0.980	0.984
	*SE*	0.0134	0.0203	0.0309	0.0126	0.0203	0.0305	0.0137	0.0259	0.0320
	Residual pattern		Scattered			Scattered			Scattered	

Note: GG-P, pure gum ghatti film; GG-G, gum ghatti film with incorporation of glycerol; GG-G-N, gum ghatti film with incorporation of glycerol and nisin.

**Table 3:** Characteristics parameters for enthalpy–entropy relationships for sorption of GG-based films.

Samples	*T*_β_ (*K*)	Δ*G*_β_ (kJ/mol)	*R* ^2^
GG-P	359.3	535.0	0.999
GG-G	332.4	598.6	0.999
GG-G-N	335.2	1031.6	0.999

Note: GG-P, pure gum ghatti film; GG-G, gum ghatti film with incorporation of glycerol; GG-G-N, gum ghatti film with incorporation of glycerol and nisin.
